# Buttermilk as Encapsulating Agent: Effect of Ultra-High-Pressure Homogenization on Chia Oil-in-Water Liquid Emulsion Formulations for Spray Drying

**DOI:** 10.3390/foods10051059

**Published:** 2021-05-11

**Authors:** Fatemeh Aghababaei, Mary Cano-Sarabia, Antonio J. Trujillo, Joan M. Quevedo, Victoria Ferragut

**Affiliations:** 1 Centre d’Innovació, Recerca i Transferència en Tecnologia dels Aliments (CIRTTA), TECNIO-UAB, XIA, Departament de Ciència Animal i dels Aliments, UAB-Campus, Universitat Autònoma de Barcelona, 08193 Bellaterra, Spain; fatemeh.aghababaei@e-campus.uab.cat (F.A.); toni.trujillo@uab.cat (A.J.T.); 2 Catalan Institute of Nanoscience and Nanotechnology (ICN2), CSIC and The Barcelona Institute of Science and Technology (ICN2), UAB-Campus, 08193 Bellaterra, Spain; mary.cano@icn2.cat; 3 Servei Planta de Tecnologia dels Aliments (SPTA), UAB-Campus, Universitat Autònoma de Barcelona, 08193 Bellaterra, Spain; joanmiquel.quevedo@uab.es

**Keywords:** buttermilk, chia oil, high-pressure homogenization, oil-in-water emulsions, spray-dried emulsions

## Abstract

Functional foods are highly demanded by consumers. Omega-3 rich oil and commercial buttermilk (BM), as functional components, used in combination to produce emulsions for further drying may facilitate the incorporation to foods. Ultra-high-pressure homogenization (UHPH) has a great potential for technological and nutritional aspects in emulsions production. The present study aimed to examine the potential improvement of UHPH technology in producing buttermilk-stabilized omega-3 rich emulsions (BME) for further drying, compared with conventional homogenization. Oil-in-water emulsions formulated with 10% chia: sunflower oil (50:50); 30% maltodextrin and 4 to 7% buttermilk were obtained by using conventional homogenization at 30 MPa and UHPH at 100 and 200 MPa. Particle size analysis, rheological evaluation, colloidal stability, zeta-potential measurement, and microstructure observations were performed in the BME. Subsequent spray drying of emulsions were made. As preliminary approximation for evaluating differences in the homogenization technology applied, encapsulation efficiency and morphological characteristics of on spray-dried emulsions (SDE) containing 21.3 to 22.7% oil content (dry basis) were selected. This study addresses the improvement in stability of BME treated by UHPH when compared to conventional homogenization and the beneficial consequences in encapsulation efficiency and morphology of SDE.

## 1. Introduction

Consumer tendency to make food choice is greatly based on their healthy characteristics. Those tailored functional foods contain added bioactive ingredients, with a physiological function in the human organism [1]. Within the functional foods offered in the market, there are protective foods for preventing chronic diseases and/or improving some natural protective systems’ development. The functional ingredients of interest include a wide range of bioactive components. Among those, omega-3 and some phospholipids have health-promoting activities.

Buttermilk (BM) is the liquid fraction obtained from butter production. It is largely considered as a by-product in the dairy industry, usually incorporated into feed. However, when processed by concentration and spray drying, it is used as an ingredient in food formulations as a partial substitute of milk solids. BM is rich in milk fat globule membrane (MFGM) residues. The MFGM is the natural encapsulating system of milk fat, consisting in a triple layer of phospholipids and proteins, so that it has a great potential as a functional ingredient. In food emulsions, the proteins and phospholipids content of BM has a techno-functional role as stabilizing agents due to their surface-active properties, which adsorbed at the interface oil-water create a viscoelastic layer preventing from coalescence [2]. From a bio-functional point of view, gangliosides, polar lipids and proteins of the MFGM have been described as health-promoting components by preventing infections, improving immunity, protecting and supporting adequate growth in healthy infants, and improving brain and cognitive system development [3,4,5]. At the same time, the use of BM as encapsulating agent, gives a great opportunity for revaluating this by-product of the dairy industry and also providing benefits from an environmental point of view by reducing its waste.

It has been confirmed the potential of BM as a new ingredient for encapsulation in atomized emulsions compared to milk proteins, and as a delivery system for bioactive compounds, such as rich fatty acids (FA) omega-3 oils [6,7]. The use of whole BM stream is an opportunity for higher value applications in functional food by capitalizing the dual functionality of BM, i.e., techno-functional and physiological functionality [6]. In this sense, the commercial BM could be a valuable ingredient for multiple applications.

Chia (*Salvia hispanica* L.) seed oil is a vegetable source with a high content of omega-3 FA, with up to 67.8% of the total lipid fraction [8]. Consumption of omega-3 FA must be incorporated to the human diet since they are essential, and are involved in multiple physiological regulations and protection against heart disease, among others [9]. In the last revision of FAO/WHO report [10] for consuming recommendation of fats and fatty acids, omega-3 FA dietary intake recommendations were established between 0.5 and 2% of the total fat intake to prevent coronary heart diseases. The use of oils high in linolenic acid such as chia oil is an interesting tool to increase the contribution of omega-3 FA to the diet.

The most common way to incorporate a lipophilic compound to food products is by emulsions encapsulation [11]. Therefore, emulsions consisting in omega-3 FA rich oils encapsulated by buttermilk surface active compounds is a potential way to incorporate those bioactive substances to produce functional foods.

Emulsions are unstable thermodynamic colloidal systems, which exhibit destabilization phenomena such as creaming, aggregation, and coalescence. These phenomena, despite an adequate formulation, take place to some extent during storage. In addition to the quality of emulsifiers, one of the most effective mechanisms to stabilize emulsions is the reduction of the droplet size in terms of both thermodynamic stability, by decreasing the attractive interaction of droplets, and kinetics, by delaying the creaming destabilizing process. To produce stable emulsions, high-energy mechanical devices, such as high-pressure homogenizers or sonication equipment, are used at industry. These homogenizers create intense breakdown forces to reduce the size of the pre-emulsion oil droplets. Ultra-high-pressure homogenization (UHPH) is a versatile technology that has the ability to inactivate microorganisms and enzymes, to give rise to submicron emulsions of great physical stability, and to produce some modifications in colloidal structures, due to the high pressures applied ranged from 100 to 350 MPa [12,13]. UHPH have a potential to produce restructuring of the protective layer of the droplets according to the composition of the emulsifying agents used, with repercussions on the techno-functional properties. Several studies [13,14,15,16] performed in emulsions using this technology have demonstrated the improvement of colloidal stability when compared to conventional homogenization.

To make easier handling, transport, and preservation for a longer time of emulsions with encapsulated functional compounds, a common practice is dehydration. Spray drying is one of the most used techniques for the microencapsulation of oils due to the high availability of equipment and low production costs compared to the other methods. Obtaining a homogeneous and stable BME previously to spray drying is decisive [17], and it is directly related to a reduced droplet size distribution, ideally submicronic [18].

This study aims to investigate the ability of commercial BM to produce BME suitable for further spray drying. It is intended to compare the emulsion characteristics of conventional high-pressure homogenization with UHPH treatment for elucidating the ability of this technology for encapsulating omega-3 oil with BM. The drying of emulsions previously processed by UHPH has not been studied yet, thus it is hypothesized that those BME could lead to improved characteristics of powders obtained. For this purpose, this study was mainly focused in the BME characteristics and, in less extent, in those obtained by spray drying.

## 2. Materials and Methods

### 2.1. Materials

Maltodextrin (MD) Glucidex^®^ 19-Maltodextrin was purchased from Roquette Freres (Lestrem, France) with 19 DE. Buttermilk powder (BM) had the following composition provided by the company: 30% protein, 7% fat, 52% lactose, less than 4% moisture and 7% ash, and was purchased from Activa Food-Tech, S. A. (Girona, Spain). Crude Chia oil (20% C-18:2, >56% C-18:3 according to the specifications) was obtained from Interfat Natural Oils (Barcelona, Spain). Crude Sunflower oil (4–9% C-16:0, 1–7% C18:0, 15–85% C18:1, 50–72% C18:2) was purchased from Gustav Heess Oils (Barcelona, Spain). All other chemical used were of analytical or better grade.

### 2.2. Emulsion Preparation

Four formulations of oil-in-water emulsions were prepared with an oil mixture of chia and sunflower (50:50), MD as wall material and BM as emulsifier. Continuous phase of emulsions was prepared by dispersing individually MD (50% *w*/*w*) and BM (30% *w*/*w*) with a Thermomix (Vorwek, Wuppertal, Germany) at 2000 rpm for 5 min. The resulting aqueous dispersions were stored overnight at 4 °C for complete hydration. To prepare pre-emulsions, the corresponding weights of MD dispersion to final concentration of 30% (*w*/*w*), BM dispersions (to 4–7% *w*/*w*) and water were mixed. Oil was slowly added to the pre-warmed (40 °C) aqueous phase and stirred by using a conventional rotor-stator mixer (Charles Ross & Son Company, Hauppauge, NY, USA) at 15,000 rpm for 5 min. The final solid content of the emulsions varied from 44 to 47% (*w*/*w*). Coarse emulsions were further homogenized using conventional or UHPH treatments at 40 °C inlet temperature. Conventional homogenization (CH) of pre-emulsions was performed in a benchtop Homolab (FBF Italia, Sala Baganza PR, Italy) at 30 MPa. Subsequently, heat treatment of emulsions was made at 65 °C, 30 min. UHPH treatments at 100 and 200 MPa were processed in an Ypsicon equipment Model A-60, which is a high-pressure continuous device (60 L/h) (Ypsicon Advance Technologies, S.L., Barcelona, Spain) that works up to 300 MPa. Working temperatures of samples were 40 °C inlet, 60 ± 2 and 80 ± 3 °C, respectively to 100 and 200 MPa at the high-pressure valve, and 25 °C outlet temperature, reached after a quick cooling by a heat exchanger connected to the UHPH equipment. Residence time in the high-pressure valve was less than one second. Emulsions were collected in Pyrex bottles for further sampling and analysis. In Table 1, the designation and composition of emulsions produced and analyzed are detailed.

### 2.3. Emulsion Characterization

#### 2.3.1. Particle Size Analysis

Particle size and distribution of emulsions were measured in the fresh samples after UHPH or CH treatments using a Mastersizer laser diffraction 2000 analyzer (Malvern Instruments Ltd., Worcestershire, UK). Emulsion were added directly to the recirculating measuring cell containing distilled water or 0.5% SDS solution until 5–9% obscuration was achieved. The optical model based on the Mie theory of light scattering by spherical particles was used. The optical model used were a refractive index of 1.460 for BM, a refractive index of 1.332 for water, and absorption of 0.01. Results were expressed as the volume-weighted mean diameter (d_4.3_, μm) and Span index. Measurements were made separately for water and SDS as dispersing media. Span is a parameter which indicates the homogeneity of the particle size distribution and was calculated using Equation (1).

Span = (d_90_ − d_10_)/d_50_ (1)

where d_x_ (μm) is the size point below which x% of the particles is contained.

#### 2.3.2. Zeta Potential

The zeta-potential of emulsions was measured using a Zetasizer Nano-ZS (Malvern Instruments, Worcestershire, UK). Emulsions were diluted 1:100 with ultrapure water and allowed to equilibrate at 25 °C for 120 s in the cuvette prior to analysis. The measurement was performed on the same day of homogenization using an automatic voltage selection. Zeta-potential was calculated using the Smoluchowski model using the software provided by Malvern Instruments.

#### 2.3.3. Rheological Evaluation

Flow curves were performed on fresh emulsions (24 h after treatment) at 20 °C with a controlled stress rheometer (Haake Rheo Stress 1, Thermo Electron Corporation, Karlsruhe, Germany). A concentric cylinders probe was used. Samples were loaded into the probe for 5 min before starting the test in order to reach equilibrium.

Flow curves were obtained in ascending and descending shear rates in the range of 0.1 and 100 s^−1^ for 60 s, respectively. Ostwald de Waele rheological model (Equation (2)) were fitted for descending curves, and the rheological parameters (K, n) were obtained. From the difference between the area under the ascendant and descendant curves, the hysteresis was calculated as indicative of thixotropic behavior.

σ = Kγ^n^ (2)

where s is the shear stress (Pa), K is the consistency index (Pa.s^n^), g is the shear rate s^−1^, and n is the flow behavior index (n = 1 indicates Newtonian behavior n ≠ 0 indicates non-Newtonian behavior).

#### 2.3.4. Physical Stability

Colloidal stability of the emulsions was evaluated by using Turbiscan MA 2000 optical analyzer device (Formulaction, Toulouse, France). Emulsions were transferred into borosilicate glass tubes of 27.5 mm dimeter up to 40 mm height, and sodium azide (0.04%) was added to prevent microbial growth. Three tubes of each sample were prepared and stored at 20 °C for 8 days. The evolution of stability was analyzed at 0, 1, 4, 6, and 8 days. This equipment provides a powerful technique for characterization of dispersions, detecting variations in stability phenomena by measuring backscattering (BS) along the sample tubes. Stability index (TSI, Formula 3) and creaming layer thickness evolution was determined by using the software (Turbisoft 2.3.1.125 version) provided by the manufacturer.

(3) $$\mathrm{TSI}=\frac{\sum h\left[{\mathrm{scan}}_{i}\left(h\right)-{\mathrm{scan}}_{i-1}\left(h\right)\right]}{H}$$

where *scan*_i_ (h) is mean BS for each i of measurement, *scan*_i−1_ (h) is mean BS for i−1 measurement, and H is the height of a sample. Higher TSI indicates stronger destabilization caused by particle aggregation and/or dynamic migration.

#### 2.3.5. Confocal Observations

A confocal laser-scanning microscope (Leica TCS SP5, Leica Microsystems GmHB, Mannheim, Germany) was used to observe the structure of fresh emulsions (24 h after production). The protein and oil were fluorescently labelled together and separately from phospholipid components of the samples. Proteins were labelled by Fast Green FCF (Sigma-Aldrich, St. Louis, MO, USA), prepared at concentration of 1% in distilled water and subsequent addition of 10% to the emulsion, excited by a 633 nm laser, and detected at 650–750 nm. Oil was labelled by Nile Red (5H-Benzo-phenoxazine-5-one, 9-diethylamino; Sigma-Aldrich, St. Louis, MO, USA), by dissolving 1 mg/mL in acetone. About 100 μL of Nile Red solution was added to 1 mL of emulsion. Excitation was made at a 488 nm laser and detected at 500–600 nm. Phospholipids were labelled by Liss Rhod PE (1,2-dioleoyl-sn-glycero-3- phosphoethanolamine-N-lissamine rhodamine B sulfonyl; 1 mg/mL; Avanti Polar Lipids Inc., St. Louis, MO, USA), by adding 40 μL to 1 mL emulsion, excited by a 561 nm laser and detected at 575–630 nm. All fluorescently labelled samples were mounted on cavity plates and examined at room temperature with a 100× oil immersion objective.

### 2.4. Spray Drying

BME were dried in a Mini Spray-Dryer B-290 (Büchi Labortechnik AG, Flawil, Switzerland). The samples were tempered at 25 °C, and the drying working conditions were 150 °C inlet temperature, 80% aspiration, and 30% feed flow. To improve conservation and reduce possible oxidative damage, the solid samples were collected in aluminum bags that were heat-sealed and stored at –80 °C for further analysis.

### 2.5. Microstructure of SDE

The morphology of the solid emulsions was observed by SEM, using the Quanta ™ 650 FEG scanning electron microscope (FEI Company, Hillsboro, OR, USA), with an accelerating beam voltage (HV) of 5 kV. Samples were prepared by fixing a small amount of powder on metal discs with double-sided carbon tapes, which were then platinum-plated in a Leica EM ACE600 vacuum chamber (Leica Microsystems, Wetzlar, Germany).

### 2.6. Encapsulation Efficiency

Extraction of free oil from SDE was carried out as described by Gonzalez et al. [19]. Dried emulsions (2.00 ± 0.01 g) were weighed and transferred to a beaker containing 30 mL of petroleum ether, stirred for 1 min and filtered. The filter paper was washed with 10 mL of petroleum ether through a pre-weighed flask to evaporate organic solvent under vacuum. Finally, the flask was heated at 105 °C in an oven to constant weight for 1 h. Encapsulation efficiency (EE) was determined according to Equation (4).

(4) $$\mathrm{EE}=\frac{\left[\mathrm{TO}-\mathrm{SO}\right]}{\mathrm{TO}}\times 100$$

where, TO is the total oil contained in the microcapsules, and SO is the free oil on surface.

### 2.7. Statistical Analysis

Results are presented as mean ± standard deviation. All data were subjected to a one-way analysis of variance (ANOVA) test using the Minitab Express™ version 1.5.3 (Minitab, State College, PA, USA). Significant differences between means were determined by Tukey test. A confidence level of 95% (*p* < 0.05) was used. At least two individual productions of each formulation and treatments were performed. All analysis were replicated three times.

## 3. Results and Discussion

### 3.1. BME Characterization

#### 3.1.1. Particle Size and Distribution

**Figure 1:**
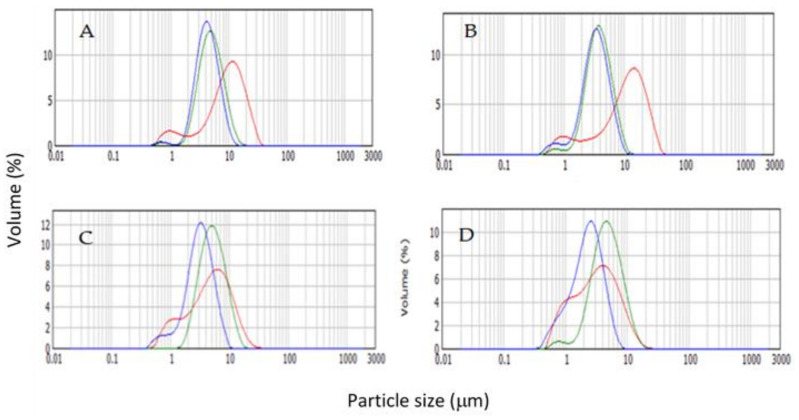
Particle size distribution curves measured by laser diffraction of emulsions containing different BM concentration: (**A**) 4%, (**B**) 5%, (**C**) 6% and (**D**) 7%, processed by CH (red), UHPH at 100 MPa (green) and UHPH at 200 MPa (blue).

The particle size distribution of BM stabilized emulsions is shown in Figure 1. In Table 2, the volume weighted (d_4.3_) mean values and Span index for emulsions dispersed in water or 0.5% SDS, respectively, are shown.

A bimodal distribution was observed in most of samples represented by a small population of particles in the range of 0.5–1 μm and a big population, which varied in function of BM concentration and homogenization conditions applied. The exception to these distribution curves were the UHPH processed emulsions with 7% BM at 200 MPa, which shifted to a unimodal distribution. The span values indicated narrower peaks of UHPH-treated emulsions compared to CH, indicating a higher homogeneity in the particle size of the former emulsions. In most of BM concentrations formulations, the CH processed emulsions showed higher particle size than UHPH emulsions. D_4.3_ values of CH-processed emulsions decreased as BM concentration increased from 4 to 7%, although differences only were significant (*p* < 0.05) between 4–5 and 6–7% BM concentrations. In UHPH-treated emulsions, increasing pressure from 100 to 200 MPa caused a reduction in d_4.3_ within the same BM concentration, although not always significant. The most accused reduction of d_4.3_ in UHPH treatments was observed in UH200-treated samples as BM concentration increased from 4 to 7%.

When comparing d_4.3_ values of emulsions dispersed in water and in 0.5% SDS, droplet aggregation phenomena was revealed. Values of d_4.3_ emulsions dispersed in SDS were considerably lower than those dispersed in water in all cases, which was caused by the breakdown of aggregates by the dispersing agent. In the case of UHPH-processed emulsions, particle size of individual droplets (SDS dispersed droplets) is in agreement with those reported by Fernandez-Avila et al. [20], who studied UHPH-treated emulsions prepared with soy protein at 4% and 10% soy oil. In this study, particle size of oil droplets in emulsions processed at 100 and 200 MPa were of 0.3 and 0.4 μm, respectively, which correspond to values found in the present study for formulations with 6 and 7% BM processed at 100 and 200 MPa.

The aggregate formation in UHPH-treated emulsions [15] prepared with sodium caseinate and sunflower oil, and several vegetable beverages, such as tigernut, almond, and soy [21,22,23], have also been described. In the mentioned studies, the presence of these structures were bigger and more abundant as pressure increased from 100 to 300 MPa, and increasing inlet temperature of samples. Authors attributed the aggregation formation to partial protein denaturation caused by the combined effect of high pressure and temperature increase in the high-pressure valve during UHPH treatment. Inlet temperature of emulsions in UHPH treatments in this study was of 40 ± 3 °C, which rise to 60 ± 4 and 80 ± 3 °C at 100 and 200 MPa, respectively, at the pressure valve, for less than one second. Samples were quickly cold down at 25 ± 2 °C in the heat exchanger connected to the UHPH equipment. Thus, these UHPH conditions were not especially conducive to the aggregate formation. Apart from forces acting in UHPH treatments, responsible for partial denaturation and disintegration of whey proteins and casein micelles, respectively, aggregate formation could be explained by a combined effect of factors. On the one hand, depletion flocculation could be the most possible mechanism of aggregation due to the effective reduction of individual droplets in the UHPH treatments [24]. Moreover, the presence of 30% of MD in the formulations could have contributed to droplet aggregation, probably due to the thermodynamic incompatibility between carbohydrates and proteins [25].

#### 3.1.2. Microstructure

BM composition provides techno-functional components involved in the formation and stability of emulsions. Milk proteins, especially caseins, and polar lipids from MFGM are the surface-active components of this ingredient. Polar lipids contain ionic groups, exerting a repulsive force between oil droplets. Proteins stabilize emulsions by forming a viscoelastic layer at the oil–water interphase. The effect of this adsorbed layer on emulsion stability combines electrostatic and steric repulsion, preventing from coalescence. In foods containing both polar lipids and proteins, binding each other through electrostatic and hydrophobic interactions [26] may take place. Thus, combined layers of polar lipids and proteins from BM are the most likely oil droplets protection of these emulsions.

**Figure 2:**
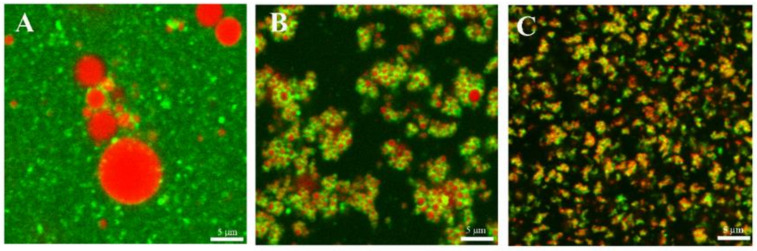
CLSM images of emulsions containing 5% BM showing the oil droplets aggregates: (**A**) 5CH, (**B**) 5UH100 and (**C**) 5 UH200. Scale bar 5 μm. Oil (red) stained with Nile Red; protein (green) stained with Fast Green FCF.

The CLSM images (Figure 2) of freshly prepared emulsions were obtained with selective staining. On the one hand preparations stained to visualize the lipid (red) and protein (green) structures together were made. On the other hand, preparations were stained to observe the polar lipids (cyan). Images showed the difference in microstructure between CH and UHPH emulsions. A background of protein (green) was observed in CH emulsions, which had a lower surface area of droplets to be protected by surface-active components compared to UHPH-treated emulsions. The presence of aggregates in UHPH-processed emulsions was observed in all formulations independently of BM concentration (not shown). In those emulsions, proteins can be seen located as part of aggregates formed by small oil droplets and acting as bonding material between them.

**Figure 3:**
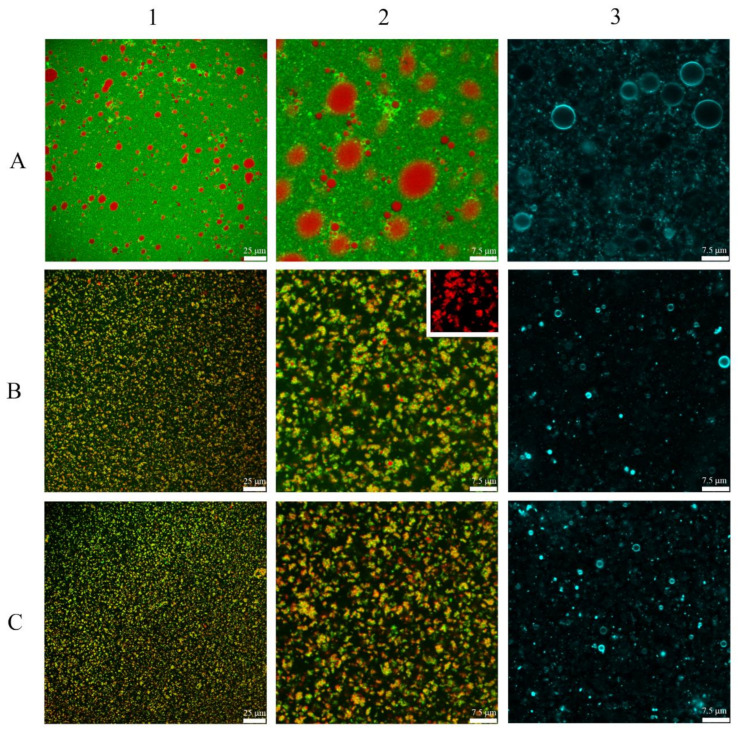
CLSM images of emulsions containing 7% BM. Columns 1 (scale bar 25 μm) and 2 (scale bar 7.5 μm) are images of the overlay of green labelled protein (stained with Fast Green FCF) and red labelled fat (stained with Nile Red). Column 3 (scale bar 7.5 μm) corresponds to images of cyan labelled polar lipids labelled images (stained with Liss Rhod PE). (**A**) 7CH, (**B**) 7UH100, and (**C**) 7UH200. (**B2**) Detail of fat labelled aggregates.

The difference in aggregates size and arrangement between droplets produced in emulsions is noticeable (Figure 3). While in CH emulsions the attachment within droplets appeared weak, in UHPH-treated emulsions, there were considerably lower sizes than the previous ones, and the attachment of droplets into the aggregates appeared tightly joined. At 100 MPa, those structures were bigger with irregular shape, while at 200 MPa, a smaller size and regular shape were observed, constituting a more homogeneous disperse phase than those emulsions treated at 100 MPa. This pattern was observed in all BM concentration emulsions.

Images of column 3 in Figure 3, polar lipids distribution around oil droplets can be observed. All images showed a good and homogeneous coverage of droplets. From images, it seems that at least a high percentage of oil droplets are well protected by polar lipids, which probably interacted with proteins to create a combined oil droplets protection.

#### 3.1.3. Rheological Behavior

Flow curves of emulsions were fitted to the Power law and rheological parameters K (consistency index) and n (flow behavior index) of emulsions for descendant curves are shown in Table 3. Flow curves and viscosity curves (Supplementary Materials Figures S1 and S2, respectively) analysis revealed a non-Newtonian behavior with slight thixotropic character in all emulsions. All samples, to a greater or lesser extent, exhibited a low variation of apparent viscosity values as shear rate increased during shearing. However, time dependence of shearing varied from one to another samples, especially depending on homogenization treatment applied, and to a lesser extent, according to BM concentration. This behavior demonstrates a certain degree of structuration of emulsions due to interactions between colloidal particles, i.e., oil droplets and BM proteins. The no coincidence of ascendant and descendant shearing curves observed were especially evident at low deformations values in CH and UH200 (from 0 to approximately 30 s^−1^) independently of BM concentration of formulation. UH100 samples showed a more marked thixotropic character, with hysteresis area being detected nearly in all range of shear rate applied in the flow curves. Hebishy et al. [27], found a Newtonian behavior in emulsions with 5% sodium caseinate and 10% oil when treated by UHPH or conventional homogenization. The presence of aggregates in the present formulations, and probably the contribution of 30% MD in the continuous phase are responsible of the thixotropic character of emulsions, contributing to a certain degree of structuration of the samples.

Consistency index and flow behavior index were calculated only in the descendant flow curve to assimilate the flow conditions during most of the operations at industry. K values of samples ranged from 0.02 to 0.09 Pa.s in all formulations. Although results did not reveal a clear pattern within samples in relation to homogenization treatment and BM concentration, some general behavior could be observed attributable to the presence of aggregates. Since viscosity manifestation depends to a great extent not only on particle size, but also on asymmetry of particles, aggregates could be the reason for the observed results. Aggregate formation and destruction do not follow a pattern since they are formed randomly. Thus, the particles shape and their asymmetry may vary to some extent in each individual emulsion production and during shearing. This could be the reason that caused a variability of frictional response during rheological measurements of emulsions with a partial disintegration during shearing. It could be hypothesized that aggregates formed in CH emulsions are easily disintegrated during shearing since those are weakly packed, as can be seen in Figure 3. Thus, in the descendent curves, K value of CH and UH200 emulsions were closer in most of cases. It could be attributed to particle size. In UH200 emulsions, aggregate sizes were closer to the individual droplets of CH emulsions, as shown in d_4.3_ (SDS) in Table 1. We assumed that the shearing of UH200 emulsions was not sufficient to breakdown those aggregates, as hysteresis was observed in UH200 samples which showed the lowest values. Thus, viscosity manifestation in these emulsions could be attributed to the friction of small and tightly packed homogeneous aggregates. However, UH100 emulsions exhibited the highest K and hysteresis values. As mentioned previously, these samples had bigger aggregate sizes than UH200. Probably the pressure difference of UHPH from 100 to 200 MPa is responsible for different degrees in casein micelles disintegration and conformation [12] in both 100 and 200 UHPH treatments, producing the observed differences in aggregate size and interaction forces. In this sense, during shearing of UH100 emulsions, the manifestation of higher K values and hysteresis may be due to a partial destruction of aggregates, which provoked a higher structuration degree and internal friction during flowing.

In any case, the viscosity values in all emulsion formulations ranged in values far from the maximum suitable for spray dried, being the final objective of these emulsions. Di Battista et al. [28] confirmed that to obtain a good atomization, viscosity of emulsion has to be lower than 0.3 Pa.s.

#### 3.1.4. Physical Stability

In this study, the influence of homogenization treatments and BM concentration was evaluated by using Turbiscan^®^ equipment during 8 days of storage at 20 °C. The backscattering (BS) at t = 0 was considered as reference to analyze the stability evolution of the system. The evolution curves during monitoring of samples by obtaining the difference of backscattering (ΔBS) gives information about the destabilization phenomena such as creaming, flocculation and coalesce, which are the common phenomena occurring in emulsions during storage. The reduction of BS in the bottom of the vial containing the sample and the increase of BS values in the top area were observed in all the ΔBS profiles (Supplementary Materials Figure S3) of samples, indicating that creaming was the main destabilization mechanism in these emulsions.

**Figure 4:**
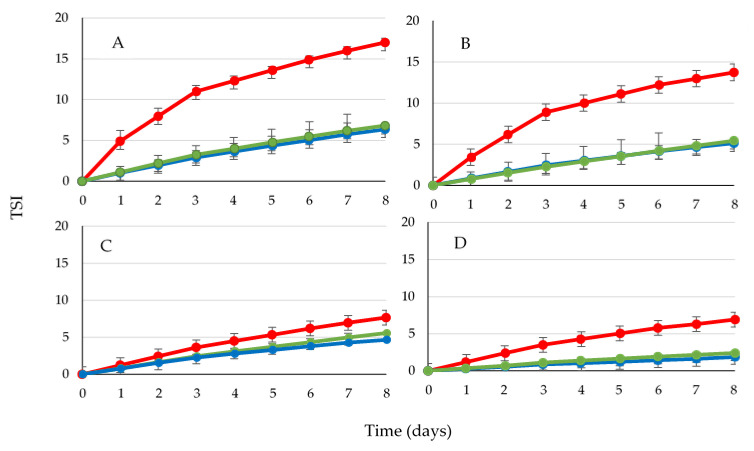
Evolution during 8 days storage of stability index (TSI) of emulsions with different BM concentration and homogenization treatments: (**A**) 4% BM; (**B**) 5% BM; (**C**) 6% BM; (**D**) 7% BM. CH (red line); UH100 (green line); UH200 (blue line).

The velocity of migration of the oil droplets and aggregates toward the surface is mainly determined by their particle size, the viscosity of the continuous phase, and by the density difference between the continuous and the disperse phases [26]. From the evolution of ΔBS profiles during eight days, a global stability index (TSI, Figure 4) and creaming layer thickness (CL) at day 1 and day 8 (Table 4) weret calculated.

CH emulsions always presented higher values of TSI than UHPH samples, indicating that the former were less stable than the latter. UHPH samples barely showed differences in stability between 100 and 200 MPa. With the same tendency, the effect of BM concentration, was significant. At 4 and 5% of BM, the TSI difference was more marked between CH and UHPH samples than in 6 and 7% BM emulsions. Related to the CL values (Table 4), the results were concomitant with TSI values. No significant differences (*p* < 0.05) were found between UHPH treatments, while they were significant as compared to the CH emulsions. Although the influence of BM concentration on CL in UHPH treatments was not significant statistically (*p* < 0.05), there was a correlation of creaming layer thickness with the type and degree of homogenization applied, which in turn was in line with the particle size values observed (Table 2). The presence of big aggregates in the CH samples and the flocculation phenomenon observed during the storage, which was observed in the ΔBS curves (Supplementary Materials Figure S3), explain the high tendency of these samples towards creaming compared to UHPH-treated emulsions. In the ΔBS curves, the flocculation phenomenon is observed if there is separation of the profiles in the middle of the vial and the different days of monitoring. In UHPH emulsions, the aggregation phenomena was found in all formulations from the beginning; however, aggregates behaved as small particles with limited mobility to the top. Flocculation was not observed during the storage in any of UHPH samples as the ΔBS profiles showed (Supplementary Materials Figure S3).

In addition to the type of homogenization applied, the BM concentration appeared as the main factor in TSI index and CL values. Increasing BM from 4–5 to 6–7% might increase the amount of adsorbed protein at the interface, with a closer packing layer formation resulting in higher stability [29].

Zeta-potential is the charge that develops at the interface between particles and the continuous, and it is used to evaluate the colloidal stability in dispersed systems. Zeta-potential is also related to the ionized groups from protein layers that surround oil droplets, when the pH was above the isoelectric point [30]. Values of zeta-potential were in the range from −22 to −29, which are near, but not in the optimum range values considered to have good stability due to electrostatically repulsion (i.e., higher/lesser than ±30 mV). Thus, the stability of these emulsions by electrostatic repulsion was not the most relevant contribution. There was no correlation of zeta-potential with homogenization procedure, nor with BM concentration. This variability in zeta-potential values might be related to the heterogeneous distribution of proteins into the aggregates observed, especially in UHPH emulsions, in which, zeta-potential values had less negative charge. Probably, the protein was occluded inside the aggregates, hindering the exposure to the surface of those colloidal structures.

### 3.2. Spray-Dried Emulsions

#### 3.2.1. Encapsulation Efficiency

**Figure 5:**
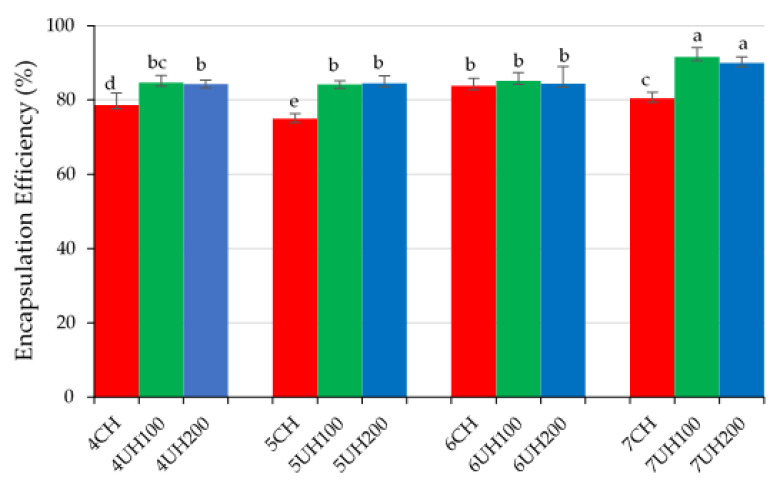
Encapsulation efficiency of the SDE from the different homogenization treatments (CH, UHPH at 100 MPa and UHPH at 200 MPa) applied to the BME, containing different percentages (*w*/*w*) of BM (4, 5, 6 and 7%). Different letters indicate significant differences (*p* < 0.05).

The degree to which wall material can prevent the internal oil extraction is given by the encapsulation efficiency (EE) [31]. This is an important parameter taken into consideration for the quality of dried emulsions. In Figure 5, comparison of the SDE with different formulations and homogenization treatments applied to BME can be seen.

**Figure 6:**
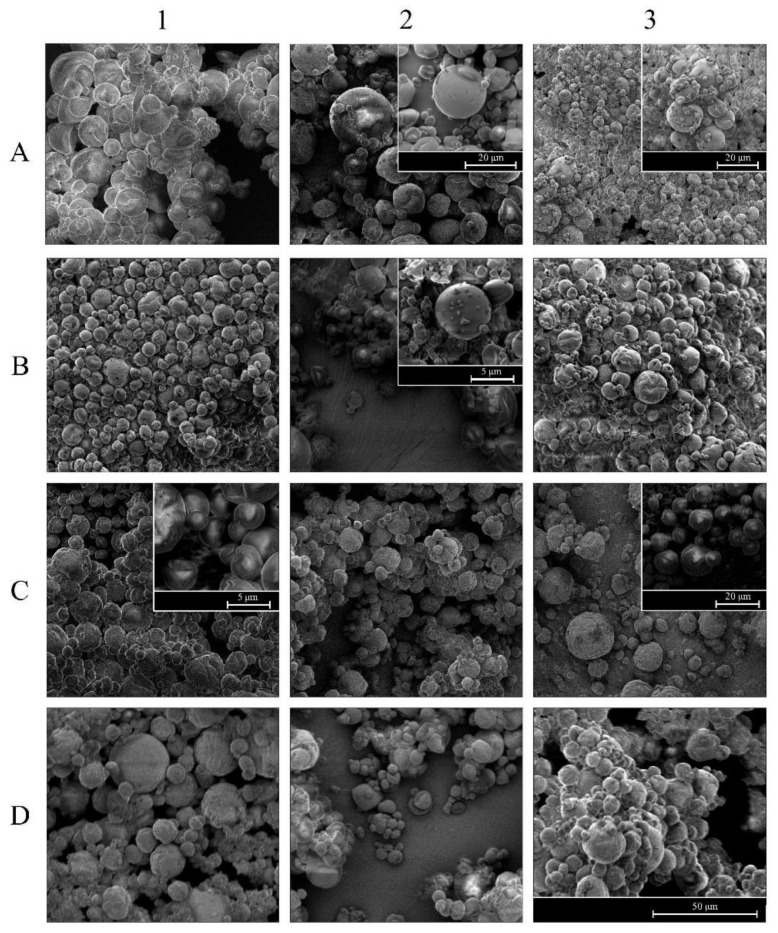
SEM images (2691×) of emulsions obtained by the different homogenization treatments: (**1**) CH; (**2**) UHPH at 100 MPa; (**3**) UHPH at 200 MPa, with different BM concentration: (**A**) 4% BM; (**B**) 5% BM; (**C**) 6% BM; (**D**) 7% BM. Magnification of overlapped images are: (**A2**) 6177×; (**A3**) 4974×; (**B2**) 9767×; (**C1**) 20614×; (**C3**) 5267×.

The main formulation factors affecting encapsulation efficiency are total solid content and the ratio of oil to wall material and oil content. In the present emulsions, total solid content varied from 44 to 47%, and oil content was from 21.3 to 22.7% (dry basis) corresponding to formulations with 4 to 7% BM. MD content remained constant (30%) in all emulsions. The corresponding ratio of oil to wall material varied from 1: 3.4 to 1: 3.7 according to the increase of BM content. In agreement to previous studies reported by Botrel et al. [32], the optimum for encapsulation efficiency in terms of mass ratio of oil to wall material could be 1:3 and less than 1:4 (*w*/*w*). Thus, the present formulations are in range. Values of free oil on the surface of powder particles decreased, in general, as total BM concentration increased for all samples. Within the same BM content, the homogenization technology applied significantly (*p* < 0.05) affected the free oil on the surface, which was always higher in CH emulsions, indicating the positive effect of UHPH technology on the encapsulation efficiency (Figure 6). However, the pressure intensity applied in UHPH produced similar results. A significant improvement of encapsulation efficiency was observed in formulations with 7% BM, which had around 90% EE. Especially in those emulsions, the difference between CH and UHPH was more marked, indicating that probably the colloidal structures created in the UHPH processing could be responsible for maintaining a better oil encapsulation. The small oil droplets attached by proteins in the aggregates would create a protective system for oil migration to the surface. BM containing casein, whey protein, and phospholipids has good emulsifying properties contributing to oil protection. Zhang et al. [7] reported encapsulation efficiency values between 89.6 and 94.3% in SDE formulated with BM, with or without MD as wall materials. Although the formulations differed substantially in BM content from that of the present study, those authors attributed to BM a great oil encapsulating capacity and found that increasing MD from 33 to 66.7% considerably decreased the encapsulation efficiency.

#### 3.2.2. Morphology

The morphological structure of the obtained microcapsules after the spray-drying of emulsions was examined by scanning electron microscopy. The SEM images of the dried emulsions are illustrated in Figure 6. All the images showed microcapsules with similar aspect, always being spherical, without apparent fractures, and with more or less rough surfaces without noticeable differences between the powders with different percentages of BM or homogenization treatments.

In all the SDE, heterogeneity in particle sizes could be observed, ranging from 5 to 20 μm, clearly distinguishing at least two populations of different sizes. This size variety has also been observed in different studies [33,34], and is a typical characteristic presented by particles that have been spray dried.

Depression and superficial folds were observed in some microcapsules; this may be due to the sudden contraction that occurs in the early stages of drying. The viscoelastic properties of emulsifiers influence the appearance of these folds [32], which may be due, in this study, to the presence of MD in the formulation of the emulsions. Korma et al. [35] observed that emulsions formulated with whey proteins and maltodextrin had a higher number of dents than those formulated with whey proteins alone.

In dry emulsions obtained by conventional homogenization [6], formulated with MD and BM at different concentrations, the integrity of the microcapsules observed in the SEM images of this study indicated that BM had good properties to form a film, which efficiently protected seaweed oil contained in capsules.

## 4. Conclusions

From this study, it can be concluded that commercial BM could be applied to stabilize emulsions with omega-3 oil for further spray drying. The application of UHPH technology improved the global quality characteristics of BME in terms of stability. These formulations, especially the UHPH-processed emulsions, induced the aggregation of oil droplets when combining MD and BM. However, the particle size of the colloidal structures in the UHPH-treated emulsions was similar or lower than the individual oil droplets produced by conventional homogenization. The presence of aggregates seems to be relevant in the rheological behavior of all emulsions studied, with a slight thixotropic character, although the equilibrium viscosity at relative low deformations showed adequate values for spray drying purposes. Results of SDE obtained to evaluate the influence of UHPH technology applied to the BME showed good behavior in terms of powder morphology and encapsulating efficiency. The formulation containing 7% BM presented the best general characteristics in both BME and SDE.

## Figures and Tables

**Table 1:** Name of samples and formulation composition of initial emulsions.

Sample Name	H (MPa)	Oil% (*w*/*w*)	MD% (*w*/*w*)	BM% (*w*/*w*)	TS% (*w*/*w*)
4CH	30	10	30	4	44
4UH100	100	10	30	4	44
4UH200	200	10	30	4	44
5CH	30	10	30	5	45
5UH100	100	10	30	5	45
5UH200	200	10	30	5	45
6CH	30	10	30	6	46
6UH100	100	10	30	6	46
6UH200	200	10	30	6	46
7CH	30	10	30	7	47
7UH100	100	10	30	7	47
7UH200	200	10	30	7	47

CH (emulsions processed with conventional homogenizer); UH (emulsions processed with ultra-high-pressure homogenizer); H (homogenization pressure); Oil (50:50, chia:sunflower); MD (maltodrextrin); BM (buttermilk); TS (total solids).

**Table 2:** Droplet size parameters (d_4.3_ and Span index) of fresh emulsions (24 h after production) dispersed in water (w) and SDS.

Emulsions	d_4.3_ (w) (μm)	Span (w)	d_4.3_ (SDS) (μm)	Span (SDS)
4CH	13.1 ± 2.50 ^a^	1.8 ± 0.100 ^bcd^	2.60 ± 0.500 ^b^	1.9 ± 0.300 ^b^
4UH100	5.45 ± 0.02 ^bc^	1.27 ± 0.01 ^e^	0.69 ± 0.070 ^d^	1.00 ± 0.04 ^cd^
4UH200	4.64 ± 0.03 ^bcd^	1.19 ± 0.02 ^e^	0.495 ± 0.008 ^d^	1.00 ± 0.04 ^cd^
5CH	11.5 ± 0.90 ^a^	2.04 ± 0.02 ^bc^	2.60 ± 0.400 ^b^	1.7 ± 0.100 ^b^
5UH100	4.16 ± 0.05 ^bc^	1.2 ± 0.100 ^e^	0.56 ± 0.020 ^d^	1.15 ± 0.01 ^c^
5UH200	3.65 ± 0.04 ^de^	1.35 ± 0.02 ^de^	0.40 ± 0.010 ^d^	0.89 ± 0.06 ^cd^
6CH	5.93 ± 0.10 ^b^	2.21 ± 0.01 ^b^	1.92 ± 0.010 ^c^	1.7 ± 0.100 ^b^
6UH100	5.08 ± 0.10 ^bcd^	1.39 ± 0.04 ^de^	0.42 ± 0.040 ^d^	0.9 ± 0.100 ^cd^
6UH200	3.44 ± 0.03 ^de^	2.0 ± 0.600 ^bc^	0.32 ± 0.010 ^d^	0.75 ± 0.03 ^d^
7CH	4.5 ± 0.500 ^bcd^	2.7 ± 0.300 ^a^	3.9 ± 0.8000 ^a^	2.5 ± 0.400 ^a^
7UH100	4.4 ± 0.900 ^bcd^	1.59 ± 0.09 ^cde^	0.43 ± 0.010 ^d^	0.83 ± 0.03 ^cd^
7UH200	2.48 ± 0.08 ^e^	1.61 ± 0.09 ^cde^	0.30 ± 0.010 ^d^	0.73 ± 0.03 ^d^

Means with different letters in the same column are significantly different at *p* < 0.05.

**Table 3:** Rheological parameters (consistency index, K, and flow behavior index, n) of descendant flow curves, and the hysteresis area from flow curves of fresh emulsions (24 h after production).

Emulsions	K (Pa.s ^n^)	n	Hysteresis (Pa/s)
4CH	0.07 ± 0.010 ^abc^	0.96 ± 0.0100 ^cd^	5.9 ± 1.20 ^cd^
4UH100	0.07 ± 0.020 ^abc^	0.96 ± 0.0100 ^cd^	10.7 ± 1.1 ^b^
4UH200	0.045 ± 0.007 ^c^	0.980 ± 0.007 ^bcd^	3.4 ± 0.10 ^d^
5CH	0.042 ± 0.003 ^e^	0.970 ± 0.009 ^cd^	4.4 ± 0.60 ^d^
5UH100	0.06 ± 0.0100 ^abc^	0.98 ± 0.0200 ^bcd^	7.8 ± 2.10 ^bc^
5UH200	0.04 ± 0.0100 ^de^	1.002 ± 0.001 ^ab^	3.5 ± 0.20 ^d^
6CH	0.045 ± 0.007 ^de^	0.985 ± 0.004 ^abc^	5.1 ± 0.60 ^cd^
6UH100	0.083 ± 0.003 ^ab^	0.95 ± 0.0100 ^d^	10.1 ± 0.2 ^b^
6UH200	0.05 ± 0.0100 ^de^	0.97 ± 0.0100 ^cd^	4.9 ± 0.10 ^cd^
7CH	0.057 ± 0.006 ^cde^	0.981 ± 0.002 ^abcd^	6.2 ± 0.90 ^cd^
7UH100	0.09 ± 0.0100 ^a^	0.974 ± 0.003 ^bcd^	14.7 ± 3.5 ^a^
7UH200	0.043 ± 0.003 ^e^	1.00 ± 0.0100 ^a^	5.5 ± 0.10 ^cd^

Means with different letters in the same column are significantly different at *p* < 0.05.

**Table 4:** Stability parameters (creaming layer, CL) of fresh (1 day) and stored emulsions (8 days) from backscattering measurements curves, and zeta-potential of fresh emulsions (1 day).

Emulsions	CL-d1 (mm)	CL-d8 (mm)	Zeta-Potential (mV)
4CH	3.8 ± 0.4 ^a^	9.7 ± 0.9 ^a^	−26 ± 1 ^bcd^
4UH100	0.8 ± 0.3 ^c^	3.3 ± 0.6 ^b^	−29 ± 2 ^d^
4UH200	0.7 ± 0.3 ^c^	2.8 ± 0.4 ^b^	−22 ± 1 ^a^
5CH	4.2 ± 1.0 ^a^	12 ± 3.0 ^a^	−28 ± 1 ^cd^
5UH100	0.8 ± 0.4 ^c^	4.4 ± 0.8 ^b^	−23 ± 1 ^ab^
5UH200	0.7 ± 0.2 ^c^	4.2 ± 0.5 ^b^	−25 ± 1 ^abcd^
6CH	1.3 ± 0.8 ^bc^	5.1 ± 2.0 ^b^	−27 ± 2 ^bcd^
6UH100	1.0 ± 0.1 ^c^	4.4 ± 1.0 ^b^	−23 ± 1 ^abc^
6UH200	0.7 ± 0.2 ^c^	3.1 ± 0.4 ^b^	−28 ± 1 ^abcd^
7CH	2.2 ± 0.400 ^b^	4.8 ± 2.0 ^b^	−27 ± 4 ^cd^
7UH100	0.53 ± 0.05 ^c^	2.3 ± 0.4 ^b^	−25 ± 1 ^abc^
7UH200	0.38 ± 0.08 ^c^	2.1 ± 0.6 ^b^	−25 ± 1 ^abc^

Means with different letters in the same column are significantly different at *p* < 0.05.

## Data Availability

Data is contained within the article or Supplementary Materials.

## Supplementary Materials

The following are available online at https://www.mdpi.com/article/10.3390/foods10051059/s1, Figure S1: Flow curves of emulsions (4 to 7% BM, 10% oil and 30% MD) processed by CH and UHPH. Figure S2: Viscosity curves of emulsions (4 to 7% BM, 10% oil and 30% MD) processed by CH and UHPH. Figure S3: Backscattering profiles of emulsions (4 to 7% BM, 10% oil and 30% MD) processed by CH and UHPH during storage.
