# Supplementary material for: Buttermilk as Encapsulating Agent: Effect of Ultra-High-Pressure Homogenization on Chia Oil-in-Water Liquid Emulsion Formulations for Spray Drying
Source: Foods. 2021 May 11;10(5):1059. doi: 10.3390/foods10051059 (PMC8151354; doi:10.3390/foods10051059)
Supplement: Supplementary file 1 [file foods-10-01059-s001.zip › foods-1208682-supplementary.pdf]

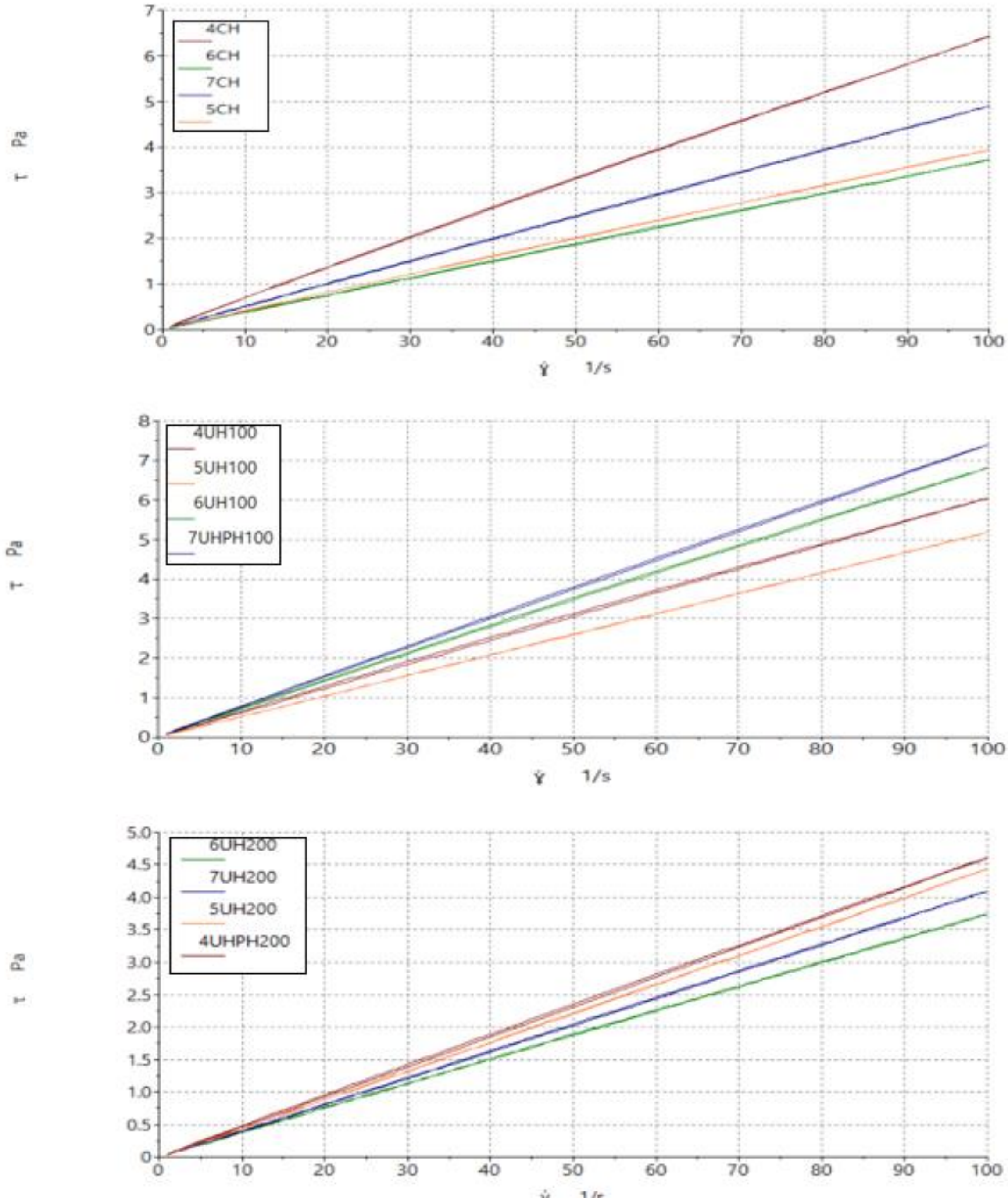

Figure 1. Flow curves of emulsions (4 to 7% BM, 10% oil and 30% MD) processed by CH and UHPH (100 and 200 MPa).

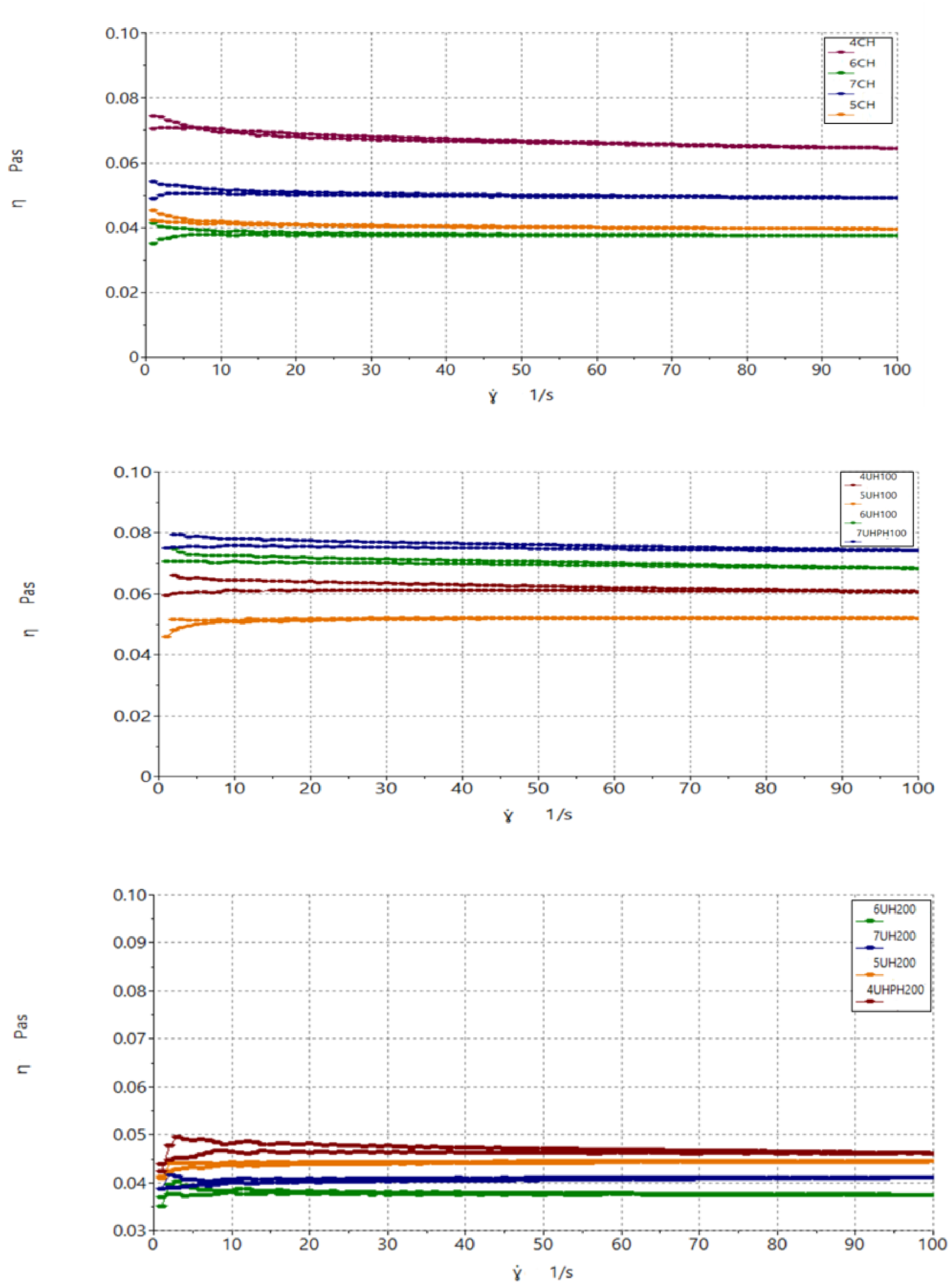

Figure 2. Viscosity curves of emulsions (4 to 7% BM, 10% oil and 30% MD) processed by CH and UHPH (100 and 200 MPa).

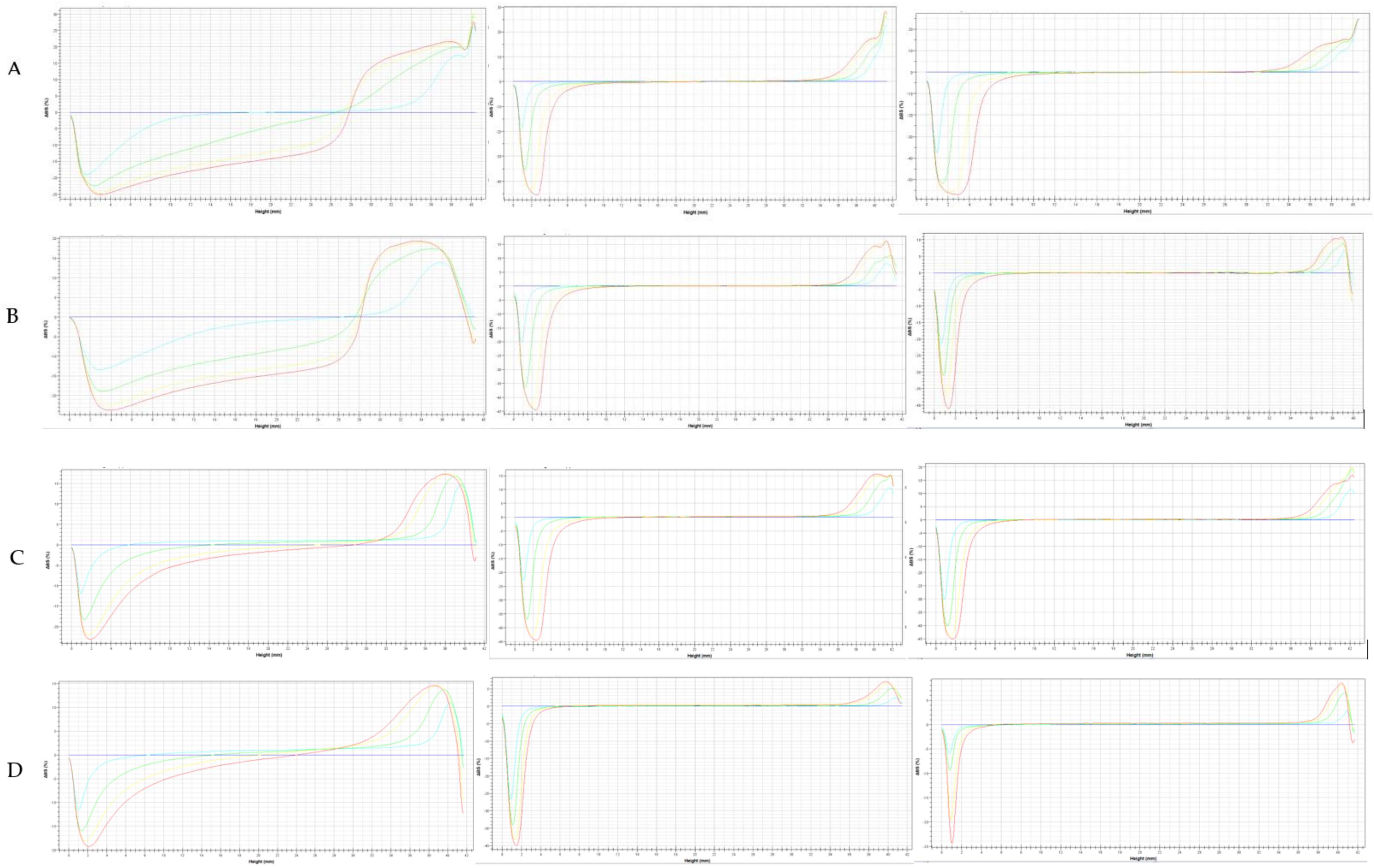

Figure 3. Backscattering profiles (ΔBS) of emulsions (4 to 7% BM, 10% oil and 30% MD) processed by CH and UHPH (100 and 200 MPa). **A**: 4CH, 4UH100, 4 UH200; **B**: 5CH, 5UH100, 5UH200; **C**: 6CH, 6UH100, 6UH200; **D**: 7CH, 7UH100, 7UH200. Blue line (day 0, reference); Red line (day 8).
